# Low-level laser therapy as a treatment for chronic pain

**DOI:** 10.3389/fphys.2014.00306

**Published:** 2014-08-19

**Authors:** J. Derek Kingsley, Timothy Demchak, Reed Mathis

**Affiliations:** ^1^Human Performance and Autonomic Studies Laboratory, Department of Exercise Physiology, Kent State UniversityKent, OH, USA; ^2^Department of Applied Medicine, Indiana State UniversityTerre Haute, IN, USA; ^3^Reed Mathis DCTallahassee, FL, USA

**Keywords:** fibromyalgia, myofascial pain syndrome, class IIIb laser, analgesia

Chronic pain is defined as pain that persists for greater than 12 weeks (Task-Force, 1994) and currently affects roughly 30% of the population in the United States (Johannes et al., 2010). The most common method for managing chronic pain has traditionally been pharmacological (Nalamachu, 2013). These treatments often include non-steroidal anti-inflammatory drugs (NSAIDS), opioids, acetaminophen, and anticonvulsants (Nalamachu, 2013). Alternative medicine is now also being used more frequently to treat chronic pain and may consist of acupuncture (McKee et al., 2013), Tai Chi (Wang et al., 2010; Wang, 2012), and low-level laser therapy (LLLT) (Enwemeka et al., 2004; Ay et al., 2010). The focus of this manuscript is to highlight the physiological aspects of LLLT, and to discuss its application for those suffering from chronic pain, alone and in combination with exercise. It will also provide justification for the use of LLLT using specific data and case studies from the existing literature which have resulted in positive outcomes for those suffering from chronic pain.

The physiological mechanisms of LLLT are not well-understood and the mechanisms tend to be very broad (Yamamoto et al., 1988; Kudoh et al., 1989; Campana et al., 1993; Sakurai et al., 2000; Chow et al., 2007; Moriyama et al., 2009; Cidral-Filho et al., 2014). One hypothesis is that there may be an increase in nociceptive threshold after LLLT resulting in neural blockade, specifically an inhibition of A and C neural fibers (Kudoh et al., 1989; Chow et al., 2007). This inhibition may be mediated by altering the axonal flow (Chow et al., 2007) or by inhibiting neural enzymes (Kudoh et al., 1989). In addition, data suggests an increase in endorphin production (Yamamoto et al., 1988) and opioid-receptor binding via opioid-containing leukocytes with LLLT (Cidral-Filho et al., 2014). LLLT may also mimic the effects of anti-inflammatory drugs by attenuating levels of prostaglandin-2 (PGE2) (Campana et al., 1993) and inhibiting cyclooxygenase-2 (COX-2) (Sakurai et al., 2000). In addition, data have suggested that LLLT may augment levels of nitric oxide, a powerful vasodilator, which would in turn act to increase blood flow and assist with healing (Samoilova et al., 2008; Moriyama et al., 2009; Cidral-Filho et al., 2014; Mitchell and Mack, 2013). While the mechanisms have not been completely explained, it is clear that LLLT may have an analgesic effect.

Studies have demonstrated that LLLT may have positive effects on symptomology associated with chronic pain (Fulop et al., 2010; Hsieh and Lee, 2013); however this finding is not universal (Ay et al., 2010). A meta-analysis utilizing 52 effect sizes from 22 articles on LLLT and pain from Fulop et al. (2010) demonstrated an overall effect size of 0.84. This would be classified as a large effect size and suggests a strong inclination for the use of LLLT to reduce chronic pain. Twenty-two studies were utilized with doses ranging from 1 to 30 J/cm^2^. On the other hand, a meta-analysis from Gam et al. (1993) demonstrated no effect of LLLT on musculoskeletal pain but this study was published over 20 years ago when LLLT was just emerging. More recently data from Ay et al. (2010) have reported no difference in chronic pain compared to placebo using twice weekly treatment 5 days a week for 3 weeks. Treatment consisted of a total energy of 40 J/cm^2^ (850 nm, 100 mV, a treatment spot area of 0.07 cm^2^, 4 min over each of the four different points). Taken together, it is hard to assess whether LLLT is an effective modality. However, it is clear that LLLT may be effective in treating chronic pain in many individuals and should not be overlooked as a treatment modality.

A systematic review and meta-analysis from 16 randomized control studies on LLLT and neck pain (Chow et al., 2009) interpreted the analysis that LLLT caused an immediate decrease in pain for acute neck pain and up to 22 weeks post in chronic neck pain patients. Recently, in a double blinded placebo control study Leal et al. (2014) reported a decrease pain and increase in function in patients with knee pain.

One issue with these meta-analyses is that participants were grouped together, under the heading of chronic pain. However, chronic pain has different manifestations which inhibit the ability to make general observations. Separate subheadings of chronic pain may include but are not limited to chronic neck pain and lower back pain, myofascial pain syndrome, and fibromyalgia. A meta-analysis by Gross et al. (2013) worked to separate out the effect of LLLT on a variety of different conditions. Based on their review, the effect of LLLT on chronic neck pain has a moderate level of evidence for effectiveness when using 830 or 940 nm but not 632.8 nm. However, it was mentioned that the trials investigating chronic neck pain and LLLT failed to blind participants which may limit the application of the data. The authors also included the effect of LLLT on myofascial pain syndrome and reported that the data are mixed and evidence is lacking. In addition, LLLT treatments have been reported to be effective for decreasing pain and increasing function in other chronic pain pathologies including fibromyalgia syndrome (Gur et al., 2002a,b; Armagan et al., 2006; Moore and Demchak, 2012).

Studies that examine the use of LLLT combined with exercise seem to have merit, as exercise is a staple of rehabilitation. Interestingly, Djavid et al. (2007) and Gur et al. (2003) both combined LLLT with exercise and each reported no additional effect of exercise in patients with chronic lower back pain. Djavid et al. utilized 27 J/cm^2^ of total energy (810 nm, 50 mW with an aperture of 0.2211 cm^2^, 8 points total) while Gur et al. utilized 1 J/cm^2^ (10 W with an aperature of 10.1 cm^2^, 4 min per point) for each of the 8 points. Matsutani et al. (2007) combined stretching exercise with LLLT (830 nm, 30 mW with an intensity of 3 J/cm^2^ over 18 tender points) in 20 women with fibromyalgia. There was no additive effect of combining stretching with LLLT in this study. Both groups reported reductions in pain scores and fatigue. Ultimately, the data are scarce and more are needed to truly understand the implications of LLLT when combined with exercise.

What tends to plague research using LLLT as a treatment modality is that there is no standard of care. Studies differ in overall dosage and wavelength which limits the ability to accurately draw conclusions. Currently, there are also no long-term studies that have evaluated LLLT. Pain is a very complex condition that manifests itself in a variety of different forms. Perhaps there is no set standard of care that will encompass everyone's needs. However, it is clear that LLLT may be beneficial for many individuals suffering from pain, regardless of the condition that is causing it.

## Conflict of interest statement

The authors declare that the research was conducted in the absence of any commercial or financial relationships that could be construed as a potential conflict of interest.
